# Levels of PM_10_ and PM_2.5_ and Respiratory Health Impacts on School-Going Children in Kenya

**DOI:** 10.5696/2156-9614-10.27.200912

**Published:** 2020-08-19

**Authors:** Faridah Hussein Were, Godfrey Angoe Wafula, Cromwel Busolo Lukorito, Timothy K.K. Kamanu

**Affiliations:** 1 Department of Chemistry, College of Biological and Physical Sciences, University of Nairobi, Nairobi, Kenya; 2 Department of Meteorology, College of Biological and Physical Sciences, University of Nairobi, Nairobi, Kenya; 3 School of Mathematics, College of Biological and Physical Sciences, University of Nairobi, Nairobi, Kenya

**Keywords:** school children, particulate matter, indoor air quality, lung function, respiratory diseases, school environment, Kenya

## Abstract

**Background.:**

The respiratory system of children is vulnerable to exposure to particulate matter (PM) with a diameter of less than 2.5 and 10 μm (PM_2.5_ and PM_10_) or even lower.

**Objective.:**

This study assessed PM_10_ and PM_2.5_ levels and respiratory health impacts on children in schools located in an industrialized suburb in Kenya.

**Method.:**

The PM_10_ and PM_2.5_ levels were sampled from five public primary schools in Athi River Township and a control school during the wet and dry seasons. Outdoor and classroom samples were collected concurrently on an 8-hour mean during school hours on two consecutive days in each school and analyzed using gravimetric techniques. Five hundred and seventy-eight (n = 578) pupils aged 9–14 years from these schools were also evaluated for symptoms of respiratory illnesses and lung function using a questionnaire and spirometric method, respectively, during the same periods.

**Results.:**

Indoor median PM_10_ levels (μg/m^3^) ranged from 60.8–269.1 and 52.8–232.3 and PM_2.5_ values (μg/m^3^) of 17.7–52.4 and 28.5–75.5 during the dry and wet seasons, respectively. The control classrooms had significantly (p <0.05) lower median PM_10_ levels (μg/m^3^) of 5.2 and 4.2, and PM_2.5_ levels (μg/m^3^) of 3.5 and 3.0 during the respective seasons. Nearly all the classrooms in Athi River schools had PM_2.5_ and PM_10_ median levels that exceeded the World Health Organization (WHO) recommended levels. The indoor-to-outdoor ratios varied from 0.35–1.40 and 0.80–2.40 for PM_10_ and 0.30–0.80 and 0.80–1.40 for PM_2.5_ during the dry and wet seasons, respectively, suggesting higher levels in the classrooms during the wet season. The relative risk (RR) and odds ratio (OR) presented higher prevalence of respiratory diseases following PM exposure in all the Athi River schools than the control during the dry and wet seasons. At 95% CI, the RR and OR showed strong associations between high PM_10_ and PM_2.5_ levels and lung function deficits and vice versa. The association was more prevalent during the wet season.

**Conclusions.:**

The study calls for effective indoor air management programs in school environments to reduce PM exposure and respiratory health impacts.

**Participant Consent.:**

Obtained.

**Ethics Approval.:**

The research permit and approvals were obtained from the University of Nairobi/Kenyatta National Hospital Ethics and Research Committee (KNH-UoN ERC Reference: P599/08/2016) and the National Commission for Science, Technology and Innovation (Reference: NACOSTI/P/18/4268/25724).

**Competing Interests.:**

The authors declare no competing financial interests.

## Introduction

Children spend about ten hours a day in school and more than three quarters of this time is spent in classrooms.^1^–^12^ The quality of air in classrooms is therefore an important parameter when evaluating the exposure of children to air pollutants. Particulate matter (PM) is the totality of all hazardous solid and liquid particles suspended in the atmosphere and contributes to deleterious effects on the respiratory system.^1^,^4^,^5^,^8^,^9^,^11^,^13^ Children are vulnerable to PM with diameters less than 2.5 μm (PM_2.5_) and 10 μm (PM_10_) or even lower that persist in the breathing zone.^1^,^4^–^14^ Particulate matter with diameters less than 10 μm are capable of penetrating into the upper respiratory tract, leading to adverse health effects.^1^,^4^–^9^,^11^,^13^–^15^ On the other hand, PM_2.5_ can penetrate deep into the lungs and cause infections in the lower respiratory tract.^4^,^5^,^8^,^9^,^12^–^15^

Irreversible inflammations in the respiratory and cardiovascular systems, including decline in lung function, have been reported in school-going children exposed to high levels of PM.^5^,^7^–^15^ Children are usually more active as a result of their natural exploratory tendencies and have significantly higher oxygen demand, narrow airways, lower breathing zones and under-developed biological systems.^1^–^13^,^15^ Immature detoxification mechanisms also predispose children to PM exposures.^8^,^9^,^13^,^15^ Franklin *et al.* considered the health impact of PM exposure and recommended that children and those with pre-existing cardiopulmonary diseases including the elderly should limit their time spent outdoors on days when the PM levels are elevated.^16^ Other studies have shown that ambient air pollution may have direct influence on indoor air quality depending on the airflow regime and installed ventilation mechanism.^1^,^2^–^11^,^14^ Children are more likely to be exposed to varying levels of PM from diverse microenvironments that they frequently visit. Assessing actual levels of PM in these microenvironments and correlating them to health outcomes in low resourced areas can nonetheless be a daunting challenge.^9^,^17^ Studies have, however, observed a strong correlation between personal exposure to PM and those of indoor air, but no association has been established with outdoor air.^1^,^18^,^19^

Exposure to elevated levels of indoor PM have been associated with a greater respiratory health impacts in children.^1^–^12^,^15^ School children may be especially affected as schools are located adjacent to heavy industrial settings, large-scale commercial facilities and high traffic density roads and are often exposed to high levels of pollutants.^8^–^10^,^14^,^15^,^20^ These observations are of special interest in the management of the wellbeing of school-going children who are susceptible to exposure to air pollutants from such environments. Some studies have been conducted in Kenya, including that of Shilenje *et al*., who reported 24-hour mean ambient PM levels that exceeded the World Health Organization (WHO) recommended levels from sites located downwind of the Athi River Township in Machakos County.^21^ However, none of the investigations have targeted the health related effects of exposure to PM on school-going children residing in the vicinity of emission zones. This study sought to assess levels of PM_10_ and PM_2.5_ and their effects on respiratory health among school-going children in Athi River Township.

**Abbreviations**
FEF_25–75%_: Forced expiratory flow between 25th and 75th (%) of the forced vital capacity
FEV_1_: Forced expiratory volume of air in the first second
FVC: Forced vital capacity
I/O: Indoor/outdoor ratio
IAQ: Indoor air quality
LPG: Liquefied petroleum gas
OR: Odds ratio
PFT: Pulmonary function tests
PM: Particulate matter
PTFE: Polytetrafluoroethylene
RR: Relative risk
WHO: World Health Organization

## Methods

This cross-sectional field study and related investigation commenced in January 2018 and ended in July 2019, with due adherence to the standard set of research protocols. The research permit and approvals were obtained from the University of Nairobi/Kenyatta National Hospital Ethics and Research Committee (KNH-UoN ERC Reference: P599/08/2016) and the National Commission for Science, Technology and Innovation (Reference: NACOSTI/P/18/4268/25724). The participation of public primary schools in the study was authorized by the Sub-County Director of Education.

Other necessary approvals and informed consent were sought prior to the commencement of the study. Reconnaissance was subsequently instituted to identify and delineate the sampling sites (public primary schools) located in the emission zones in Athi River Township using Geographical Positioning System and observations.

### Description of the study area

**Figure 1:**
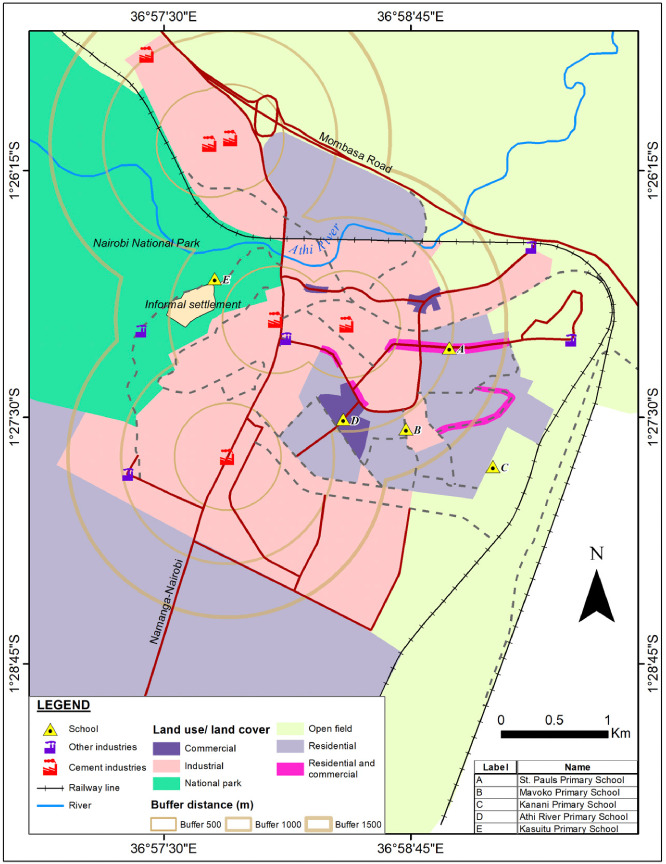
Location of sampling sites and possible emission sources in Athi River Township. Map produced by the Department of Geology of the University of Nairobi.

The study was conducted in Athi River Township of Machakos County located in the lower eastern part of Kenya, about 25 km from Nairobi County. Athi River Township is situated between latitudes 1^°^26′20″ and 1^°^27′30″ South, and longitudes 36°57′30″ and 37°0′50″ East *(Figure 1).* The topography of the township is generally flat terrain, with altitude ranging from 1,480 to 1,520 m above the sea level and covers an area of 693 km^2^. The area is designated as an industrial zone. It is characterized by a marked increase in heavy industrial activities.^21^ There are six cement-manufacturing plants *(Figure 1)* with an overall production capacity of 8 million tons of cement per annum accounting for 90% of all cement produced in Kenya. Other active industries in the area include steelworks, mining, incineration, lead acid battery recycling, salt production, long haul transport, quarrying, and related large-scale commercial activities.^15^ The sampling sites (schools) in relation to the location of the potential emission sources (industries) are shown in Figure 1.

The township is characterized by unplanned settlements that are driven by immigrants mainly comprised of unskilled and semi-skilled labor in search of employment opportunities.^22^ The road network that serves pedestrians and all types of motor vehicles is to a large extent unpaved, dusty and poorly maintained. Air pollution associated with road traffic, commercial and industrial activities presents a great health risk in the area.^21^,^22^ The township lacks fixed stations to monitor background pollutant levels.

**Figure 2:**
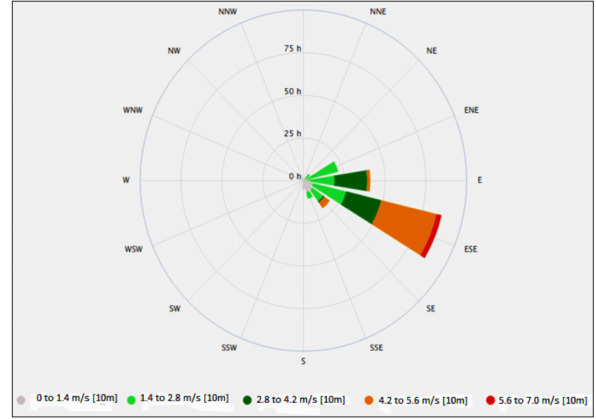
Wind rose showing the wind direction in the study area. Source: MeteoBlue^23^

Athi River Township is located in an arid and semi-arid area of Kenya, with a climate that is predominantly hot and dry throughout the year. Rainfall is usually received in the months of March to May during the long-wet season, and October to December during the short-wet season. The mean annual total rainfall is 695.4 mm. The mean minimum and maximum temperatures are 13.4°C and 25.2°C, respectively. The prevailing winds over the township are highly variable in strength and generally range from 1.3–7.0 m/s. The wind direction is predominantly east-south-easterly (ESE) as shown in Figure 2.

### Sampling and general characteristics of the sampling sites

Five schools in Athi River, coded as A, B, C, D and E *(Figure 1)* were purposively sampled on the basis of their locations relative to the nearest cement factories (between 0.6 and 2.5 km) and other industries, unpaved road traffic-related zones and commercial activities. School F, situated to the northeast of, and 40 km away from, Athi River Township (in the Kasarani area on the outskirts of Nairobi County) served as a control treatment with no known industrial or vehicular air pollution sources. Table 1 presents details of sampled schools.

**Table 1:** General Characteristics of the Sampled Schools

	**Sampled schools in Athi River Township**					**Control school**

**Parameters**	**A**	**B**	**C**	**D**	**E**	**F**
**Coordinates**						
Latitude	−1.45247	−1,45624	−1.4625	−1.45851	−1.44661	−1.22676
Longitude	36.98432	36.98448	36.9812	36.97347	36.96264	36.9108
Elevation	1541.3	1539.1	1526.1	1524	1508.7	5187
Year established	1970	2010	1997	1964	2010	2000
Total enrollment	1230	1350	922	1319	510	600
Distance from the nearest cement industry (km)	1.7	2	2.5	1	0.6	40
Nature of playfield and school grounds	Partially covered playground with few trees and patches of grass.	Bare playfield and school grounds	Exposed playground with few patches of shrubs and grass	Exposed playground with few shrubs	Exposed playground with few shrubs, grass and rocks	Paved playground, cover grass and trees
Proximity to other potential pollution sources	30 m from high density traffic tarmac road, and within the commercial and residential zones	30 m from high density traffic, unpaved road and within industrial and commercial settings	Within residential area and 50 m from unpaved road	Within commercial zones and 30 m from high density traffic on unpaved and tarmac road	Within industrial zone and 30 m from unpaved road with light traffic	Away from potential sources of pollution

### Sampling periods

Collection of samples was carried out during normal school activities that excluded weekends, examination periods and school holidays. In accordance with the government policy, the schools were also not authorized to participate or undertake any external activities during the third term (between August and October) when children were preparing for their National Examinations.^24^ This study was therefore limited to a shorter period of investigation. Data on the levels of outdoor and indoor PM_10_ and PM_2.5_ levels, symptoms of respiratory diseases together with parameters about lung function, and prevailing meteorological conditions were collected during the long rainy season, between March and May 2018 and the dry season between January and February 2019.

All the outdoor PM_10_ and PM_2.5_ levels from the six public primary schools and their corresponding classroom samples could not be simultaneously sampled due to the limited number of air samplers. The outdoor (n = 4) and corresponding classroom (n = 4) PM_10_ samples were therefore collected concurrently in an 8-hour period during the school hours on two consecutive days in each school during the wet and dry seasons. The same procedure was repeated for the sampling of outdoor and corresponding indoor PM_2.5_ during the two seasons. School E was however unable to participate in the study during the wet season. This was due to the sporadic flooding of the River Athi, which made it impossible for the researchers to gain access to the school.

### Recruitment and participation

A total of six hundred and fifty (n = 650) children were randomly recruited from 4 classrooms (class four to seven) of schools A, B, C, D and E in Athi River and the control school, F. The criteria used for recruiting pupils in this study included voluntary participation with informed consent from parents or guardians of children aged between 9 and 14 years residing within 3 km radius from their schools. We aimed for gender balance.

A survey of the participants in the six public primary schools was undertaken during the two climatic seasons using questionnaires. The subjects who were unable to participate throughout the sampling periods were excluded from the study, except for school E, which was inaccessible during the rainy season due to flooding. The design of the questionnaire followed the guidelines of the American Thoracic Society Division of Lung Diseases (ATS-DLD-78-C) questionnaire on respiratory symptoms^25^ with some modifications to maintain relevance and applicability to children. A pre-test survey was conducted prior to adoption of the questionnaire. Authorized medical officers administered the pre-tested questionnaire to participants using basic medical terms through a face-to-face interview in the presence of, and with assistance from their classroom teachers *(Supplemental Material 1).* This ensured successful completion of the questionnaires.

The survey recorded the age (years), gender, height (cm), weight (kg), and body mass index (kg/m_2_) of the participants.^24^ The medical officers then asked the respondents if they were experiencing one or more of the following symptoms: sneezing, coughing, frequent or persistent cough, wheezing, sore throat, running nose and chest pains. Persistent cough was described as experiencing frequent coughing most of the days (more than 4 times a week within the current season), and the participants were also asked if they have been medically diagnosed, hospitalized or were taking medication for related ailments including bronchitis, wheezing attacks and asthma-like symptoms within the current season. Those who were experiencing any of the symptoms were physically examined by the medical officers. The symptoms were subsequently categorized as either upper respiratory tract symptoms (sneezing, running nose, sore throat, and coughing) or lower respiratory symptoms (persistent or frequent coughs, chest pains, wheezing, shortness of breath and bronchitis). Other potential confounding factors included the type of household fuels frequently used in homes for cooking such as charcoal, firewood, liquefied petroleum gas (LPG) and paraffin, and for lighting such as electricity, candle, and paraffin. Youth aged between 9–14 years in this community are actively involved with household chores and considered knowledgeable about fuel types used and household cooking practices.

### Lung function testing

Pulmonary function tests (PFT) were undertaken to evaluate the respiratory health status of the children in the present study. The test was conducted based on the American Thoracic Society standardization of spirometry.^26^ Testing was carried out by two medical technicians trained in spirometry under the supervision of a qualified physician specialized in respiratory tract infections and approved by the Directorate of Occupational Health and Safety Services in Kenya.^27^ The exclusion criteria were those on medication for various serious ailments or any other medical conditions that could make spirometry testing unsafe or give unsatisfactory readings. The procedure for administering PFT was explained and demonstrated to the target participants in simple terms. It involved each child breathing in fully and with closed nostrils with a clip and a disposable mouthpiece tightly around the mouth to prevent air from escaping. This was followed by breathing out as fast as they could until the lungs felt empty. Each participant practiced this technique until they were able to perform the maneuvers without further instructions since the techniques depended on participant cooperation.

Lung function examinations were then performed three times with intervals of rest to avoid exhaustion. This was carried out using an electronic spirometer (Vitalograph Compact, UK), which was calibrated according to the manufacturer's specifications. This instrument measured the actual respiratory flow rate of the participants at a precision of 2%. The measured value was compared with the predicted data of lung function indices with consideration for the age, sex, height, weight, and ethnicity of each participant. The measured indices included forced vital capacity (FVC), defined as the maximum volume of air that can be forcibly exhaled in one breath after a maximum inhalation, forced expiratory volume of air in the first second (FEV_1_), which was the volume of air exhaled in the first second of the FVC maneuver, and forced expiratory flow between 25^th^ and 75^th^ (%) of the FVC (FEF_25–75%_) or the maximum midexpiratory flow. The computerized assisted assessment of the PFT indices were reviewed and the best predicted values (%) of FVC, FEV_1_, FEV_1_/FVC and FEF_25–75%_ were taken for this study. These procedures were subsequently repeated during the dry season on the same participants.

A predicted FEV_1_/FVC ratio of less than 70% of PFT was interpreted as a large airway obstruction or obstructive lung disease. The severity of ventilatory impairment was classified as mild when FEV_1_/FVC (%) was from 50–69% of PFT, moderate when it was 30–49% of PFT and severe when it was less than 30% PFT. The restrictive and small airway obstruction type of pulmonary impairment was considered when the FVC and FEF_25–75%_ were less than 80% of predicted values. The predicted % were further categorized as follows: 60–79% as mild, 40–59% as moderate and less than 40% as severe. In the combined type of impairment, both the predicted values of FVC were less than 80% and FEV_1_/FVC ratio was less than 70%.

Out of 650 pupils that were recruited to participate in the study, 72 of them could not participate because 22 were on medication for various health problems, 33 had unsatisfactory spirometric readings, and 17 failed to participate during the dry season. Five hundred and seventy-eight (n = 578) participants completed the study successfully.

### Collection of particulate matter and meteorological conditions

Portable air sampling systems, Ecotech Microvol 1100, designed to monitor indoor and outdoor PM, were used throughout the sampling periods for measuring particulate matter.^28^ The air samplers were equipped with size selective inlets with effective aerodynamic size of PM_2.5_ and PM_10_ as appropriate. Polytetrafluoroethylene (PTFE) filter membranes of 47 mm diameter, 0.2-micron pore size with support ring sequentially labeled were used. The PTFE filters were then equilibrated in a clean room in the Atmospheric Deposition Networks Laboratory, Department of Chemistry of the University of Nairobi under a temperature and humidity-controlled chamber for 24 hours. The filters were then pre-weighed 3 times using a microbalance (Mettler Toledo) with a variation of less than 5% and stored in the desiccator prior to sample collection.

The outdoor and corresponding classroom PM_10_ samples for each school were collected simultaneously in an 8-hour period, during the school day of two consecutive days during the wet season. The same procedure was followed for collection of outdoor and classroom air PM_2.5_ levels during the wet season while maintaining the same sampling points in each of the sampled schools. This was to enable the collection of representative samples of outdoor and classroom PM_10_ and PM_2.5_ levels that could have had an influence on classroom air quality during the wet season.

For the classrooms sample collections, the air samplers were centrally mounted on tripod stands, away from the walls and any form of interferences in each classroom of the participants. Inlets of 1 m off the ground level were maintained. This was taken as an approximate representative height of the average breathing zone of the participants in their sitting position. For outdoor sampling, each air sampler was placed downwind, in an east-south-easterly (ESE) direction *(Figure 2),* where the winds were predominately blowing towards the classroom. The outdoor samplers were maintained about 40 m away from each corresponding classroom at an angle between 80° and 100° with respect to the front and rear sides of the classroom, and free from any form of interferences to allow unrestricted air movement. The samplers were then mounted allowing sampling inlets of about 1.45 m off the ground, which was in conformity to the average breathing zone of the participants. This procedure was repeated during the dry season. The PM_10_ and PM_2.5_ for outdoor and classroom air samples were collected on pre-weighed filters and removed from the sampling pump by handling the filter at the metal rim using forceps to avoid contamination. The loaded filters, along with filter field blanks, were then kept in the filter cassettes in plastic containers and stored in the desiccator at the end of the sampling period.

The prevailing meteorological conditions were also monitored during the same sampling periods. The data for rainfall (mm), relative humidity (%), and wind speed (m/s) were obtained from the nearest fixed weather station located at the Jomo Kenyatta International Airport. These data sets were also augmented by observations obtained from Meteoblue.^23^

### Determination of levels of particulate matter and comparison with air quality exposure limit

The PTFE filters were post-weighed in the same laboratory following the same protocol for pre-weighing of PTFE filters. The net weight of each sample was determined by computing the difference in weight of loaded filter and pre-weighed PTFE filters and field blank filters.^29^,^30^ The concentration in microgram per cubic meter of air (μg/m^3^) were then calculated from the net weight over the total volume of air sampled for the 8-hour period.

All PM_2.5_ and PM_10_ levels (μg/m^3^) in the classrooms that were obtained were compared with the WHO indoor air quality (IAQ) guidelines for PM levels to evaluate childrens' exposure. Use of the existing WHO ambient air quality guidelines for the PM_2.5_ and PM_10_ levels of 25 μg/m^3^ and 50 μg/m^3^, respectively, in a 24-hour period against the 8-hour mean was recommended for the respective IAQ.^31^ Portugal has similar IAQ regulations for public buildings.^32^ However, the outdoor PM_2.5_ and PM_10_ levels obtained in this study cannot be compared directly with the Kenyan ambient air quality regulation of levels of 75 and 150 μg/m^3^ for the industrial sites, respectively, because of the differences in sampling periods of 8-hours versus 24-hours.^33^

### Quality control and assurance

All the analytical equipment used in this study were calibrated prior to their use as per manufacturers' instructions. Established standard operating procedures were also followed for all applicable analytical procedures. The gravimetric procedure was applied in accordance with the recommended standards for gravimetric sampling and analysis for air pollutants of PM_10_ and PM_2.5_.^28–30^ Filters were stored at a relative humidity of 45–50% and temperatures of 25–30°C in a controlled clean room for 24 hours in order to attain stable conditions.^28^ The sensitivity of the microbalance was approximately 0.001 mg. Filters were weighed in triplicates with acceptable weight variations of less than 5%. Values of flow rate were considered as 3 L per minute when they fell within 3±3% L per minute. The normal school activities were maintained throughout the sampling periods.^6^

### Data analysis

The R programming environment was used to analyze the data.^34^ Customized scripts were implemented and used to explore, wrangle, and visualize inherent characteristics between variables and calculate summary statistics. The tidyverse packages (ggplot2, magrittr, purrr, dplyr, tibble and tidyr) were used for data representation and visualization, and the epitools package was used to calculate summary statistics such as relative risks (RR), odds ratios (OR) and their associated confidence intervals.^35^,^36^ The RR and OR are relative measures of respiratory health status and are more useful and informative than absolute frequencies. In addition, RR and OR are ideal because they are marginally affected by sample sizes and have probabilistic interpretation. The calculation of RR and OR together with their corresponding confidence intervals is considered a precise and exact method based conditional Maximum Likelihood Estimation (MLE); denoted as ‘Fisher' in the results, Table 1, Supplemental Material 2. Table 2, Supplemental Material 2 presents the unconditional MLE based on large-sample (normal/Gaussian) approximations (denoted as ‘Wald') as well as normal approximations with small sample adjustments (denoted as ‘small').^37^

Exact statistical methods provided greater accuracy when calculating interval estimates as well during inference than similar asymptotic and approximate methods; exact methods are also robust with respect to sample size. Relative risks and OR were calculated to evaluate the relative magnitude, probability and risk of presenting symptoms of respiratory diseases among the exposed and non-exposed subgroups as well as to compare the strength and direction of the association between PM and ventilatory function. Relative risks were therefore taken as a ratio of the risk of exposed cases versus a similar risk associated with the control, whereas OR represented a ratio of observing the prevalence of respiratory diseases with respect to the exposed over the ratio of observing the similar outcome for the non-exposed. Relative risks and OR were then calculated in view of lung function indices as per American Thoracic Society spirometry guidelines, which provided a unified grading system for assessing the clinical relevance of the prevalence of respiratory diseases, and eliminated potential biases derived from age, height sex and ethnicity of the participants.^26^

## Results

Although there were variations in the location of the schools as shown in Figure 1 and Table 1, the design and nature of the classrooms were generally similar since they were arranged in series (blocks), running from pre-primary to upper primary (nursery to class eight). Each classroom had a cemented floor area of about 6″ × 8″ m^2^. The classrooms were roofed with iron sheets and most areas had chipped floors and cracked walls. All the sampled classrooms were naturally ventilated through open casement windows with glass panes and wooden doors of an area of about ″0.9 × 2.1″ m^2^. The windowpanes were broken in nearly all cases and hence remained opened. The mode of teaching was chalk and blackboard in all of the classrooms. Each classroom accommodated about 50 to 57 pupils aged between 4 and 15 years. Seating arrangements involved a simple wooden desk with three pupils in a row while pre-primary pupils sat singly on a plastic chair and table arranged in a row.

### Levels of PM_10_ and PM_2.5_ in the classrooms during the dry and wet seasons

**Figure 3:**
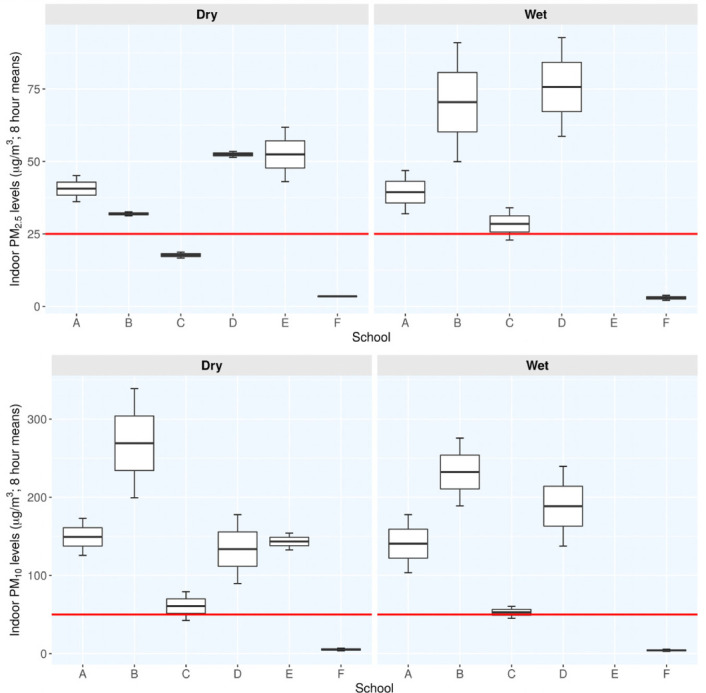
Distribution of indoor PM_10_ and PM_2.5_ levels across the schools during the dry and rainy seasons. Whiskers represent 1st and 3rd quartiles. The PM levels depicted symmetry about the median value represented by the line dividing the boxes. Indicated by the horizontal red lines, the WHO and Portuguese set limits for IAQ of 50 μg/m^3^ for PM10 and 25 μg/m^3^ for PM_2.5_ levels, respectively.^31^,^32^

Figure 3 compares the indoor levels of PM_10_ and PM_2.5_ across schools during the wet and dry seasons in an 8-hour period. Classrooms in Athi River schools (A–E) had median PM_10_ levels of 60.8–269.1 μg/m^3^ and 52.8–232.3 μg/m^3^ during the dry and wet seasons, respectively. The levels of PM_10_ did not differ significantly between the seasons (p >0.05). School F, the control, reported significantly low levels of indoor PM_10_ with values ranging from 3.5–6.9 μg/m^3^ (median 5.2; p=0.01207) and 2.8–5.6 μg/m^3^ (median 4.2; p=0.03023) compared to the schools in Athi River during the respective seasons. Overall, school B exhibited the highest indoor PM_10_ levels, with school C presenting the least levels regardless of the seasons.

The median PM_2.5_ levels in Athi River classrooms were generally lower than those of PM_10_ and varied from 17.7–52.4 μg/m^3^ and 28.5–75.7 μg/m^3^ during the dry and wet seasons, respectively. In the same way, PM_2.5_ median levels of 3.5 μg/m^3^; p=0.02105 and 3.0 μg/m^3^; p=0.02318 in the control school were significantly lower than those of the Athi River schools during the dry and wet seasons, respectively. The PM_2.5_ levels in the classrooms did not differ significantly between the seasons (p >0.05). The classrooms in school E exhibited the highest variation in the levels of PM_2.5_ during the lone sampling dry season. All the Athi River classrooms had PM_10_ and PM_2.5_ median levels that exceeded the WHO IAQ guidelines of 50 μg/m^3^ for PM_10_ and 25 μg/m^3^ for PM_2.5_, respectively, except for school C, which had a PM_2.5_ level of 17.7 μg/m^3^ that fell within the recommended limit during the dry season.

### Indoor-to-outdoor ratios of PM_10_ and PM_2.5_ levels during dry and wet seasons

**Figure 4:**
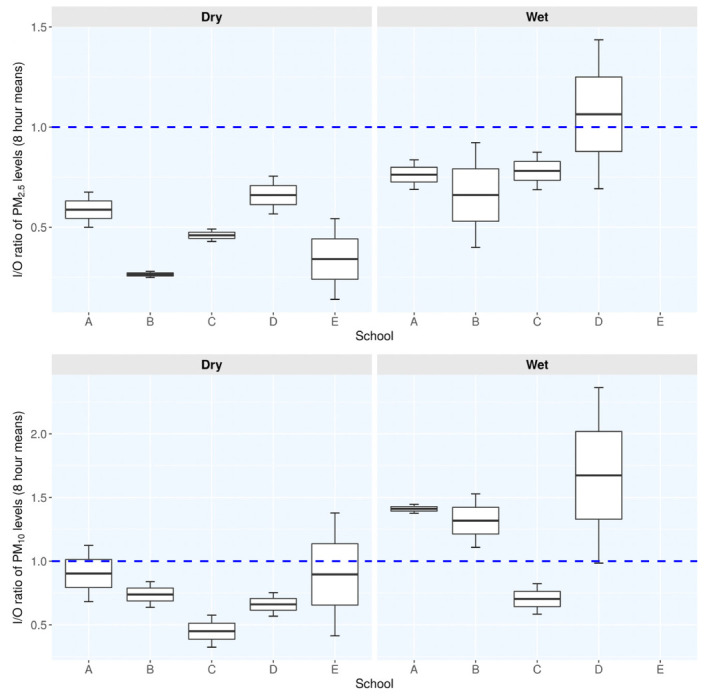
Distribution of indoor-to-outdoor ratios of PM_10_ and PM_2.5_ levels by seasons across the Athi River schools The horizontal blue dashed line indicates I/O ratio of unity (1) below which outdoor sources contribute to indoor PM levels. Values greater than 1 indicate higher levels of PM from indoor sources relative to outdoor sources.

The indoor-to-outdoor (I/O) ratios of PM_10_ levels differed from 0.35–1.40 and 0.80–2.40 during the dry and wet seasons, respectively *(Figure 4).* Outdoor PM_10_ levels were significantly higher (p=0.015) during the dry season compared to the wet season. There was a reduction of 42.1% in the PM_10_ levels following the rainfall. The I/O ratios of PM_10_ levels in schools A, B and D were greater than unity (1) during the wet season. This implies that there was additional contribution of PM_10_ levels in the classrooms over and above the outdoor source. Similar trends to those of I/O ratios of PM_10_ were observed for PM_2.5_ levels, where I/O ratios ranged from 0.30–0.80 during the dry season, with much higher ratios of 0.80–1.40 being observed during the wet season. Significantly higher (p=0.006757) outdoor PM_2.5_ levels were measured during the dry season compared to the wet season with a decrease of 28.1%.

In Athi River schools, PM_2.5_-to-PM_10_ ratios (PM_2.5_/PM_10_) ranged from 0.23–0.39 and 0.26–0.54 for the dry and wet seasons, respectively, in the indoor environment, and 0.48–0.53 and 0.48–0.70, respectively, in outdoor environments. The studied schools reported prominence of coarse particles and hence reduced PM_2.5_/PM_10_ ratios in both classrooms and outdoor environments during the dry season and increased PM_2.5_ during the rainy season. This is an indication that outdoor coarse particles are primarily being washed out of the atmosphere during the wet season. School E had the highest outdoor PM_2.5_ levels during the dry season and hence it presented the highest PM_2.5_/PM_10_ ratios, mainly fine particles, but the school did not have comparable results in the wet season.

### Demographic characteristics of the participants

**Figure 5:**
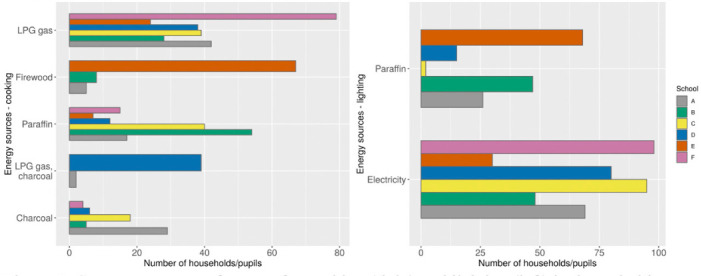
Common sources of energy for cooking (right) and lighting (left) by households across schools. Households in School F (control) mainly used clean energies for cooking (LPG gas) and lighting (electricity). Households in comprising schools A, B, C, D and E used diverse fuels including unclean sources of energy.

Five hundred and seventy-eight (n = 578) children completed the study successfully, with 95, 95, 97, 95, and 98 participants from school A, B, C, D, and E, respectively, in the Athi River area. The control school F had 98 participants and was located over 40 km from the core study area. Male and female students were equally represented in the study (p >0.05, Fisher exact test). Similarly, there were no significant variations in age and body mass index between the pupils in the schools in Athi River relative to the control school. The types of fuel used in their homes did not vary significantly during the dry and wet seasons. A few of the households in Athi River used clean fuels, including electricity and LPG. However, the majority used diverse and unclean fuels such as charcoal, firewood and paraffin for cooking and to a large extent paraffin for lighting *(Figure 5).* There were significant differences (p <0.05) observed between sources of energy for lighting and cooking in various homes of children from Athi River and the control schools. For example, a large proportion of homes of children in school E, who are residents of an informal settlement *(Figure 1),* used firewood for cooking and paraffin for lighting. Use of these sources of energy could pre-dispose and confound estimated risks from PM to the exposed group relative to the control group.

### Association of exposure to PM_10_ and PM_2.5_ with prevalence to respiratory diseases

**Figure 6:**
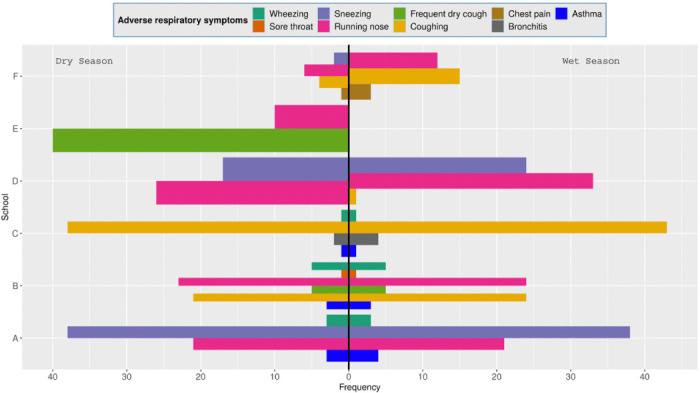
Prevalence of respiratory diseases across the studied schools during the dry and wet seasons

As illustrated in Figure 6, the participants from schools in Athi River experienced a higher prevalence of respiratory diseases that were manifested by the presence of two or more symptoms compared to the control group. This suggested greater underlying respiratory disease in children from Athi River schools. In almost all cases, the symptoms were more pronounced in magnitude during the rainy season when the PM levels in the classrooms were also elevated *(Figures 3 and 4).* More respiratory symptoms such as persistent dry or wet cough were noted in participants in school E during the dry season, with no comparable results during the wet seasons. School B had a greater spectrum of symptoms (6) compared to the other schools, with increased prevalence of upper respiratory symptoms, such as running nose and coughing and fewer cases of lower respiratory symptoms (persistent cough and wheezing). More asthmatic children were recorded in school A and B. Apart from running nose, sneezing attacks were exclusively observed in school A and D as an indication of allergic reactions. The majority of the participants in school C had upper respiratory symptoms manifested by coughing complaints and a few cases of diagnosed bronchitis. Although school C had reported relatively low levels of PM_10_ and PM_2.5_ among the Athi River schools, the PM_10_ levels exceeded the WHO recommended levels except for PM_2.5_ which was within the limit during the dry season *(Figure 3).*

**Figure 7:**
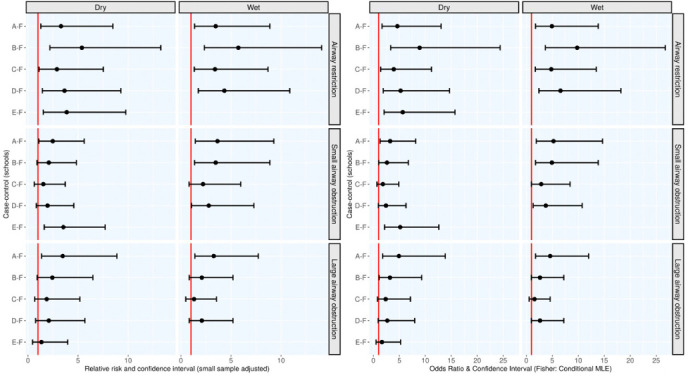
Relative risks, odds ratios, and prevalence of respiratory diseases and lung function impairment across Athi River schools (A–E) with respect to control school F. Panel columns 1, 2, 3 and 4 represent RR and OR, respectively, for schools A–E in Athi River relative to control school F by type of lung function impairment, including airway restriction (reduced FVC), small airway obstruction (reduced FEF25–75%), and large airway obstruction (decrement FEV1/FVC). The exact values of RR and OR are shown as shaded dots along the horizontal lines terminated by whiskers that mark the width of their interval estimates corresponding to 95% CI for each case school (A–E) against the control (F), and lung function abnormality indices. The null cases where RR=1 or OR=1 are indicated by a red vertical line and these imply independence between exposure and outcome variables (zero risk). School E did not participate during the wet season.

Relative risks and OR were analyzed to explore the relative extent of association between exposure to PM_10_ and PM_2.5_, and the outcomes of prevalence of respiratory diseases in Athi River with respect to the control school. Figure 7 and Table 1, Supplemental Material 2 summarized results of RR and OR and their CI at 95% calculated using the R package EpiTools. The results indicated that for all the children in Athi River schools, exposure to PM_10_ and PM_2.5_ was associated with increased risk of developing airway restriction as manifested by reduction in FVC compared to the control group during the dry and wet seasons. This is because 95% CI of both RR and OR associated with experiencing airway restriction did not overlap the null cases where RR=1 and OR=1. In particular, the probability associated with occurrence of respiratory symptoms following exposure to PM was higher than the probability of observing the same for the non-exposed. School B that had the highest levels of coarse particles among the Athi River schools also had the uppermost RR=5.38 (dry; 95% CI: 2.19–13.26), RR=5.73 (wet; 95% CI: 2.34–14.06) and OR=8.91 (dry; 95% CI: 3.20–30.95) and OR=9.79 (wet; 95% CI: 3.53–33.91) values presented for developing airway restrictions compared to schools A, C, D and E. This indicates that exposure to elevated levels of coarse particles is associated with reduction in FVC, which is a presentation of large airway restriction. In all cases, and as observed with the symptoms for respiratory diseases, airway restriction was more prevalent during the wet season compared to the dry season.

School E that had the highest PM_2.5_ levels among the Athi River schools, also had the highest RR=3.54 (95% CI: 1.62–7.71) and OR=5.16 (95% CI: 2.05– 14.83) values for small airway obstruction during the dry season. This result similarly suggests that exposure to higher PM_2.5_ levels is associated with greater RR and probability of developing small airway obstruction (reduction in the forced expiratory flow between 25^th^ and 75^th^ (%) of the FVC (FEF_25–75%_). This relationship could not be confirmed during the wet season since this school did not participate. Children in schools' B and D also had a similar risk, but only during the wet season, and these two schools had comparatively high PM_2.5_ levels in their classrooms during this period *(Figure 3).*

Nonetheless, school A had all the interval estimates of RR and OR not overlapping the null values under all types of lung function impairment. School A maintained consistently elevated levels of both PM_10_ and PM_2.5_ in classrooms regardless of the season *(Figure 3).* Children had a high likelihood of developing the combined form of pulmonary function deficit, and this risk was greater for airway obstruction as indicated by the interval estimates of RR and OR values. Allergic reactions in form of running nose and sneezing attacks were also widespread among these children *(Figure 6).*

On the contrary, school C, which had the lowest PM_10_ and PM_2.5_ exposure among the Athi River schools also had relatively low RR and OR values associated with low prevalence of respiratory diseases. This indicates that pupils at school C were not at risk of developing large and small airway obstruction associated with exposure to elevated levels of PM_2.5_, but they were at greater risk of developing airway restriction associated with exposure to elevated levels of PM_10_, as this school had PM_10_ levels that exceeded the WHO recommended levels. This is supported by RR=2.90 (dry; 95% CI: 1.11–7.52), RR=3.40 (wet; 95% CI: 1.33–8.70) and OR=3.92 (dry; 95% CI: 1.31–14.23) and OR=4.79 (wet; 95% CI:1.65–17.12) for airway restriction, which did not overlap with the null value RR=1 and OR = 1.

## Discussion

The present study showed that PM_10_ and PM_2.5_ median levels in outdoor and classroom air assessed in an 8-hour period across the Athi River schools were markedly higher than those of the control school during the two climatic seasons. The levels exceeded the WHO recommended levels in all classrooms in Athi River schools, except for school C which had PM_2.5_ levels that fell within the limit during the dry season. Overall, Athi River schools exhibited high PM levels similar to a review of studies by Oliveira *et al.* that found PM_10_ and PM_2.5_ levels (μg/m^3^) varying from 66.7–591.0 and 17.0–163.0 for indoor school environments respectively, versus 31.0–1578.0 and 15.3–242.0 for outdoor school environments, respectively, in Asian public schools.^9^

The location of the Athi River schools within the industrial and commercial zones adjacent to the unpaved road traffic could have directly influenced measured levels. Most of the industries in Athi River Township were observed to generate dense smoke and dust that varied in exposure levels. Similar studies by Shilenje *et al.* conducted downwind of these industries reported 24-hour mean ambient PM_2.5_ levels of 30.7 μg/m^3^ (range: 10.3–111.2 μg/m^3^) and PM_10_ levels of 69.6 μg/m^3^ (range: 13.5–463.3 μg/m^3^), which exceeded the WHO ambient air quality guidelines.^21^ However, in the Shilenje study, the other atmospheric pollutants including sulfur dioxide, nitrogen dioxide, ozone, carbon monoxide, hydrogen sulfide, and methane were within the recommended limits. Several other studies have reported significantly higher PM_10_ and PM_2.5_ levels in schools located in the vicinity of heavily polluted industrial complexes and high-density traffic emissions compared to non-polluting areas.^2^,^6^,^8^–^10^,^21^

Apart from industrial and commercial activities, meteorological factors also had profound effects not only on levels of outdoor PM, but also distribution of PM across the Athi River schools. The wet deposition had stronger influence on the coarse than fine particles as observed by a reduction of 42.1% of outdoor PM_10_ and 28.1% of outdoor PM_2.5_ levels during the wet season *(Figures 3 and 4).* This suggests that coarse particles were easily washed out by rain or the bulk of them formed the nucleation surfaces upon which moist air could have condensed to form cloud droplets and thereafter rained out from the atmosphere. The PM may also have been dispersed, diluted, modified, and ultimately removed by gravitational settling on the surrounding areas or ultimately washed out of the atmosphere by falling rainfall.^6^,^10^,^38^,^39^

Atmospheric turbulence in form of whirlwinds was observed to stir up previously settled dust on the ground, making it airborne. As a result of the prevailing conditions of atmospheric stability, these particles were restricted to the lower layer of the atmosphere, within the breathing zones of the children, thereby increasing their vulnerability to PM exposure. The settled particulates were further re-suspended when disturbed by various activities that involved movement of motor-vehicles and children playing, especially during the dry windy seasons. The unpaved roads with high traffic-induced particle emissions associated with land degradation in Athi River constituted potential sources of PM exposure. This clearly explains the observed variations in the I/O ratios of PM_10_ and PM_2.5_ levels from 0.35-1.40 and 0.30-0.80 during the dry and windy (4.0±0.4 m/s) seasons and 0.80–2.40 and 0.80–1.40 during the wet and calm (2.0±0.5 m/s) season, respectively. Bo *et al.* also reported lower levels of ambient PM levels for reduced wind speeds and elevated levels for stronger winds.^39^

Studies agree that ambient PM levels interact with indoor air by penetrating through open doors, windows and cracks with natural ventilation.^1^–^7^,^9^–^11^ These avenues were identified as contributing to the substantial PM levels that were measured in the classrooms across the Athi River schools during the dry season. Nevertheless, comparatively high levels of PM were found in these classrooms during the wet season. This could be explained by the dispersion of settled particles as several pupils were observed playing in their classrooms with limited ventilation when it was raining. Other studies have attributed increased indoor PM_10_ levels to resuspension processes of settled particles and slowed down deposition mechanisms due to turbulence as a result of playing activities. In addition, Fromme *et al.* reported elevated mean PM levels in classrooms compared to corresponding outdoor levels, and similar observations were made by Oeder *et al.* who found indoor PM_10_ levels were more than 5 times that of outdoor levels during teaching hours.^4^,^8^ The findings are further supported by Maryam *et al.* who reported high PM levels in classrooms near polluted environments correlated with children activities, including occupancy rate and inadequacy of ventilation.^5^

In general, several studies concur with our findings that elevated PM levels in classrooms may be prompted by resuspension of settled dust and ineffective ventilation.^1–12^ Particulate matter (PM_2.5_ and PM_10_) or smaller are the most relevant since they are capable of penetrating into the respiratory tract, causing health effects.^1^,^5^,^8^,^9^,^14^,^15^ This explains the high prevalence of respiratory diseases and increased risk of developing pulmonary function abnormalities observed in children of Athi River schools compared to the control group. These findings are in agreement with the Athi River Sub-County Annual Health Report that revealed respiratory illnesses were a common cause of consultations in community health facilities and the incidence of respiratory diseases was on the rise in the township and exceeded the national average.^40^ Other authors have reported significantly high rates of respiratory diseases that manifested through exacerbation of asthma and increased hospital visits by children in schools in close proximity to industrial areas and heavy traffic density emissions compared to those in rural areas.^1^,^4^,^6^,^8^,^9^,^1^,^15^,^20^

There were variations in the levels of PM across the studied schools by seasons, however school B consistently had the highest PM_10_ levels in the two seasons. This can be attributed to a combination of several factors such as a relatively high-density population of children on a small piece of largely bare land, with little vegetation cover. The school was close to a heavily trafficked unpaved road and children were observed kicking up already settled dust while playing and rushing to and from school. They then carried these PM in/on their shoes and clothing to their classrooms. The children were subsequently exposed to elevated levels of PM_10_ in a limited space of less than 1 m^2^ per child in their classroom. It was further observed that manufactured cement was transported by heavy trucks on the same unpaved roads that also generated and released considerable dust. All of these factors combined influenced the re-entrainment of dust by the subsequent traffic-movement and blowing winds. Atmospheric turbulences were also observed to stir up already settled particles making them airborne and able to infiltrate through open windows and doors into the classrooms.

The classrooms of school B had also the lowest ratio of PM_2.5_/PM_10_ with the highest proportion of coarse particles. Re-suspension of these particles were apparently the dominant pathway through which children were exposed to PM. In addition, a high I/O ratio was reported in classrooms of school B during the wet season, implying that playing activities could have caused re-suspension of particles since there was no specific indoor emission sources. The inherent particles were further re-suspended due to dry sweeping that took place on a Monday during the wet season when children were playing within their classrooms, and this explains the substantial indoor PM_10_ median levels of 275.7 μg/m^3^ that were measured. The re-suspension process could also have involved relatively large amounts of accumulated particles from the previous weekend. These findings are supported by those of Gui who compared dry and wet sweeping practices and found that dry sweeping resulted in increased PM levels.^41^ The study then recommended that wet sweeping under ventilated conditions could significantly reduce the levels. This explains the strong association of levels of coarse particles in school B and the high risk of developing airway restriction pulmonary abnormalities together with a spectrum of upper respiratory symptoms that were observed in children in this school. The prevalence of respiratory diseases was also more pronounced during the wet season than the dry season.

On the other hand, school E presented the highest outdoor PM_2.5_ median level of 211.1 μg/m^3^. This is a result of the predominantly east-south-easterly (ESE) wind *(Figure 2)* that blew towards school E, located downwind the direction of most industries. Studies have further found that wind speed, wind direction, atmospheric stability and rainfall have a significant impact on the diffusion, dilution, accumulation and retention of PM_2.5_.^38^ The observed PM_2.5_ level could be attributed to emissions from the cement and tannery industries since the school was in close proximity to these industries. Similar studies have found that children attending schools near cement factories among other industrial sources were more vulnerable to higher levels of PM_2.5_.^42^ Although the wind speed was positively correlated with PM, PM_2.5_ levels were reduced by 24.8% in classrooms compared to those in outdoor air. This could be due to the windows and doors of school E which were facing upwind, allowing less infiltration of particles. The high indoor PM_10_ levels shown by high SD value could be a result of children's activities that caused re-suspension of previously settled particles. The PM_2.5_ is relatively smaller in size with a larger surface area and is more easily transported than PM_10_ and penetrates deeper into the lungs, thereby having greater impact on children's health.^9^,^15^ Moreover, the present study found that children from school E had the highest proportion of lower respiratory symptoms that manifested in persistent cough with greater risk (OR=5.16; 95% CI 2.05–14.83) of developing small airway obstruction.

It is also worth noting that these children' residences were within the nearby informal settlement *(Figure 1)* and the majority of them exclusively used solid fuels (firewood) for cooking in their homes. A similar study revealed significantly high PM_2.5_ levels in the households that mainly used unclean energy source for cooking and lighting.^43^ Although the contribution of PM at the household level was not assessed, it is plausible that these children were further exposed to high levels of PM from their homes.

Schools A and D had comparable outdoor and indoor PM levels, although school D had slightly higher outdoor PM_10_ and indoor PM_2.5_ levels during the wet season, and this explains the similarities in the prevalence of respiratory diseases. A high proportion of children in both schools had upper respiratory ailments manifested in sneezing attacks and running nose. This may also explain the high number of children from school D that had restrictive lung function impairment compared to school A during the rainy season. Both schools had similar characteristics, such as population density of pupils and location within commercial zones. The relatively high levels of PM_2.5_ in classroom D could be a result of open windows and doors facing a high traffic road and also in close proximity to industries, about 1 km to the cement industry compared to the distance of 1.7 km for school A. However, it was also observed that respiratory diseases were more prevalent in school A than D and children in school A had a higher risk of developing small and large airway obstruction type of lung function impairment. Similar observations have been made that exposure to elevated indoor PM_10_ and PM_2.5_ levels was associated with increased risk of reduced FVC/FEV_1_ associated with large airway obstruction.^15^ School A had consistently high levels of PM in the classroom regardless of the season and it was also likely that children from school A were exposed to PM from household fuels as higher proportion of them used solid fuel (charcoal) for cooking and paraffin for lighting compared to school D.

School C had the lowest outdoor and indoor PM_10_ and PM_2.5_ levels among the Athi River schools, with the latter levels being within the WHO acceptable levels for the dry season. This could be attributed to the fact that school C was located furthest from the industrial sources, commercial centers, but closest to unpaved road traffic related emissions *(Figure 1 and Table 1).* Although the major focus of this study was cement manufacturing plants owing to the increase in number of plants from two to six in the recent years, we recognize that other notable emission sources such as unpaved road traffic emission, open burning of wastes, wind-blown dust due to degraded grounds, and children's play activities could be among the main drivers of increased exposure for school C. Other contributions may include household fuel. This could therefore explain the relatively lower RR and OR observed in school C, which was associated with a lower prevalence of respiratory diseases among Athi River children. However, these children were at risk of developing airway restriction and the majority had coughing complaints associated with PM_10_ levels that exceeded the WHO recommended levels. Other studies have similarly reported higher incidences of respiratory diseases and lung function deficit associated with PM exposure exceeding recommended values.^1–21^

The present study also acknowledges that the use of clean fuel was significantly higher (p <0.05) among the control group compared to Athi River households. Previous authors have reported fuel efficiencies of 20% for charcoal, 30% for wood, 50% for paraffin and 70% for LPG. The rating of fuels was significantly associated with the extent of health effects per joule of energy emitted and per socio-economic ranking.^44^ These findings are applicable to the Athi River residents where 50% of the population cannot afford or access clean fuel. It is clear from this observation that extensive use of unclean fuels could have partly contributed to PM exposure in pupils across the Athi River schools, although the actual exposure level and respiratory health impact could not be estimated. Most importantly, all the Athi River participants resided within the township, suggesting that exposure levels could be even greater than that conveyed in the present study.

### Study limitations

The general limitation of this study was the 8-hour sampling period compared to the 24-hour mean that posed a challenge for direct comparison of the results of the National Regulation for ambient air quality of 150 μg/m^3^ for PM_10_ and 75 μg/m^3^ for PM_2.5_ for the industrial sites.^33^ Actual PM_10_ and PM_2.5_ exposure levels were not determined due to lack of personal air samplers. Moreover, sampling across the schools were not conducted simultaneously due to lack of equipment and resources. The study also acknowledges the fact that indoor and outdoor PM_10_ and PM_2.5_ levels may not be the only factors responsible for adverse health outcomes in Athi River Township. Most Athi River households used various types of unclean fuel which may have a greater contribution to PM levels than what was determined, hence further studies are warranted.

## Conclusions

The present study established that PM_10_ and PM_2.5_ levels in Athi River schools that markedly exceeded those of the control school and the recommended limits posed a high risk of exposure and prevalence of respiratory diseases for schoolchildren. The levels of exposure were greatly influenced by the location of the schools, potential emission sources and seasonality. The absence of specific regulations and effective enforcement strategies means that Athi River schoolchildren continue to experience elevated PM exposure levels and adverse health related impacts. The results of the present study call for suitable interventions and strategies to monitor and control PM_10_ and PM_2.5_ related emissions at the source to avert respiratory health impact in school environments.
