# Supplementary material for: Levels of PM10 and PM2.5 and Respiratory Health Impacts on School-Going Children in Kenya
Source: J Health Pollut. 2020 Aug 19;10(27):200912. doi: 10.5696/2156-9614-10.27.200912 (PMC7453813; doi:10.5696/2156-9614-10.27.200912)
Supplement: Supplementary file 1 [file Were_Supplemental1.docx]

**Supplemental Material 1**

**PRE-TESTED QUESTIONNAIRE ON RESPIRATORY HEALTH
STATUS OF SCHOOLGOING CHILDREN**

**SEASON**………………………

**DATE**…………………………

**Instructions**

Please answer the questions as frankly and accurately as possible. All the information gathered here will be kept confidential as per the University of Nairobi/Kenyatta National Hospital Ethics and Research Committee (KNH-UoN ERC Ref: P599/08/2016) research protocol that was signed by your parents, class teachers and a medical officer.

Only recruited participants will participate in the questionnaire survey and are free to opt out at any time. The questionnaire consists of 5 parts. Information in Part I will be extracted from the Class Register by the class teacher of the recruited participant: Part II information will be gathered through measurements, Parts III and IV will be gathered through interview and Part V will involve classification of symptoms observed as either of lower or upper respiratory tract infection.

**PART 1: Background information [from the pupil’s class register]**

1. Date of Birth: [Day---------] [Month………….] [Year………….]

[Please record the pupil’s date of birth]

1. Pupil’s gender

[Please record the pupil’s gender]

1. Male
2. Female 
3. Name of the pupil’s school

[Please tick in the box the correct code of the school]

1. A
2. B 

c**)** C ****

d) D 

e) E 

f) F

1. Name of the pupil’s classroom

[Please tick in the box the correct pupil’s classroom and stream]

1. Class Four (IV)

b) Class Five V 

c) Class Six (VI) 

d) Class Seven (VII) 

1. Area of residence within three km radius from the school

[Please tick in box the correct area of residence of the pupil]

1. Athi River Township 
2. Kasarani on the outskirts of Nairobi 

**PART II: Personal information**

1. Age ……………………………years

[Please tabulate age of the pupil in years from the date of birth given in Part 1 (1.) above]

1. Body weight……………………kg

[Please take measurement of pupil’s weight without shoes]

1. Height …………………………m

[Please take measurement of height without shoes]

1. Body Mass Index (BMI) ……………..kg/m^2^

Calculate weight (kg) over height squared (m^2^) from Part II (2 and 3)

**PART III: Type of Household Fuel Used in Pupils’ Homes**

[Interview the pupil using the national language with assistance from their teacher]

1. What types of fuels are commonly used for cooking in your home/house?

a) LPG gas

b) Electricity

c) Firewood

d) Paraffin

e) I do not know

f) Any other, please specify…………………

1. What type of lighting do you mainly use when doing your homework or studies at home?

a) Electricity

b) Firewood

c) Paraffin

d) Candle

e) I do know

f) Any other, please specify……………………

**PART IV:** **Respiratory health status (prevalence of respiratory symptoms) of the pupil within the current season**

**[**Pupil interviewed by an authorized medical officer using simple medical terms in national language]

1. Within this month, have you ever suffered from one or more symptoms of respiratory infection such as sneezing attacks, coughing, common cold, sinusitis, frequent or persistent cough, wheezing, sore throat, running nose and chest pains?
2. Yes b) No
3. Any other, please specify……………...................................................
4. If yes (continue)
5. Are you experiencing cough?
6. Yes b) No

If yes (continue)

ii) How many times do you cough in a week:

a) Several times (more than 4 times a week)

b) Occasional (less than 3 times a week)

c) Once a week

d) Only today

e) I do not know

If yes continue

iii) Have you ever produced phlegm when coughing during this season?

a) Yes  b) No 

1. Have you ever coughed blood during this season?
2. Yes b) No
3. Do you experience sneezing attacks?
4. Yes b) No
5. Do you have a running nose?
6. Yes b) No
7. Do you have a sore throat?

a) Yes b) No

1. Do you frequently have a common cold?

a) Yes b) No

1. Do you experience wheezing that interferes with your schoolwork?
2. Yes b) No
3. Do you have chest pain when you breathe deeply?
4. Yes b) No
5. Is there any other problem that is related to respiratory diseases that you have suffered from/been diagnosed with?
6. Yes b) No
7. Are you currently on medication for the following conditions?
8. Breathing problems

a) Yes b) No

1. Asthma-like symptoms

a) Yes b) No

1. Bronchitis

a) Yes b) No

iv) Sneezing related to allergies

a) Yes b) No

v) Wheezing attacks

a) Yes b) No

vi) Shortness of breath

a) Yes b) No

1. Any other…………………………………………………….

If yes, continue

1. Have you ever been diagnosed with the condition you have mentioned in 11) above by a doctor/hospitalized?

a) Yes b) No

If yes continue

1. Is your class teacher aware of this condition?

a) Yes  b) No ****

**PART V:** **Classification of stated symptoms as upper or lower respiratory infection by an authorized medical officer**

Reported symptoms will be categorized as either those of the upper respiratory tract (running nose, sneezing, sore throat, and coughing) or lower respiratory tract (persistent or frequent coughs, chest pains, wheezing, shortness of breath and bronchitis).
