# Supplementary material for: Levels of PM10 and PM2.5 and Respiratory Health Impacts on School-Going Children in Kenya
Source: J Health Pollut. 2020 Aug 19;10(27):200912. doi: 10.5696/2156-9614-10.27.200912 (PMC7453813; doi:10.5696/2156-9614-10.27.200912)
Supplement: Supplementary file 2 [file Were_Supplemental2.docx]

**Supplemental Material 2**

**Table 1: Fisher representation of Odd Ratio (OR) and Relative Risk (RR) and prevalence of respiratory diseases and lung function deficits**

| **Relative Risk & Confidence Intervals (small-sample-adjusted)** | | | | | | **Fisher Odds Ratio & Confidence Intervals (Conditional MLE)** | | | | | |  |  |
| --- | --- | --- | --- | --- | --- | --- | --- | --- | --- | --- | --- | --- | --- |
| Lung function impairment | **Season** | **Dry Season** | | | **Wet Season** | | | **Dry Season** | | | **Wet Season** | | |
|  | **Compare** | **RR** | **Lower** | **Upper** | **RR** | **Lower** | **Upper** | **OR** | **Lower** | **Upper** | **OR** | **Lower** | **Upper** |
| Airway restriction | A-F | 3.300 | 1.284 | 8.480 | 3.474 | 1.359 | 8.879 | 4.615 | 1.571 | 16.565 | 4.922 | 1.688 | 17.585 |
|  | B-F | 5.384 | 2.186 | 13.262 | 5.732 | 2.337 | 14.059 | 8.913 | 3.203 | 30.948 | 9.789 | 3.534 | 33.905 |
|  | C-F | 2.892 | 1.111 | 7.528 | 3.402 | 1.330 | 8.700 | 3.926 | 1.314 | 14.234 | 4.795 | 1.646 | 17.121 |
|  | D-F | 3.647 | 1.434 | 9.278 | 4.342 | 1.734 | 10.871 | 5.236 | 1.808 | 18.632 | 6.582 | 2.320 | 23.130 |
|  | E-F | 3.872 | 1.534 | 9.773 |  |  |  | 5.657 | 1.978 | 19.971 |  |  |  |
| Small airway obstruction | A-F | 2.475 | 1.091 | 5.616 | 3.647 | 1.434 | 9.278 | 3.231 | 1.220 | 9.597 | 5.236 | 1.808 | 18.632 |
|  | B-F | 2.084 | 0.898 | 4.838 | 3.474 | 1.359 | 8.879 | 2.620 | 0.960 | 7.933 | 4.922 | 1.688 | 17.585 |
|  | C-F | 1.531 | 0.629 | 3.724 | 2.211 | 0.820 | 5.966 | 1.830 | 0.629 | 5.757 | 2.864 | 0.910 | 10.703 |
|  | D-F | 1.954 | 0.834 | 4.579 | 2.779 | 1.060 | 7.285 | 2.427 | 0.878 | 7.407 | 3.743 | 1.239 | 13.663 |
|  | E-F | 3.536 | 1.622 | 7.709 |  |  |  | 5.158 | 2.049 | 14.830 |  |  |  |
| Large airway obstruction | A-F | 3.474 | 1.359 | 8.879 | 3.275 | 1.389 | 7.720 | 4.922 | 1.688 | 17.585 | 4.586 | 1.690 | 14.566 |
|  | B-F | 2.432 | 0.911 | 6.488 | 2.084 | 0.836 | 5.197 | 3.196 | 1.031 | 11.840 | 2.637 | 0.901 | 8.778 |
|  | C-F | 1.871 | 0.675 | 5.185 | 1.312 | 0.486 | 3.546 | 2.369 | 0.723 | 9.062 | 1.565 | 0.475 | 5.576 |
|  | D-F | 2.084 | 0.763 | 5.690 | 2.084 | 0.836 | 5.197 | 2.676 | 0.834 | 10.112 | 2.637 | 0.901 | 8.778 |
|  | E-F | 1.347 | 0.457 | 3.973 |  |  |  | 1.649 | 0.456 | 6.660 |  |  |  |

There are no risks associated with lung function impairment among the children of Athi
River schools in that season since RR and OR are less than 1 (CL 95%) and there is overlapping of null cases where RR and OR =1

**Table 2: Wald representation of OR and RR and prevalence of respiratory diseases and lung function deficits**

| **Wald Relative Risk & Confidence Intervals (normal; unconditional Maximum Likelihood Estimation (MLE)** | | | | | | **PACKAGE: EPITOOLS: CALCULATION OF RELATIVE RISK (FUNCTION - RISK RATIO) Calculates risk ratio by unconditional maximum likelihood estimation (Wald), and small sample adjustment (small). Confidence intervals are calculated using normal approximation (Wald), and normal approximation with small sample adjustment (small), and bootstrap method (boot). riskratio(x,y=NULL,method=c("wald","small","boot"),conf.level=0.95,rev=c("neither","rows","columns","both")) Wald Odds Ratio & Confidence Intervals (normal; unconditional MLE)** | | | | | |  |  |
| --- | --- | --- | --- | --- | --- | --- | --- | --- | --- | --- | --- | --- | --- |
| Lung function impairment | **Season** | **Dry Season** | | | **Wet Season** | | | **Dry Season** | | | **Wet Season** | | |
|  | **Compare** | **RR** | **Lower** | **Upper** | **RR** | **Lower** | **Upper** | **OR** | **Lower** | **Upper** | **OR** | **Lower** | **Upper** |
| Airway restriction | A-F | 3.920 | 1.525 | 10.074 | 4.126 | 1.614 | 10.547 | 4.650 | 1.659 | 13.034 | 4.960 | 1.778 | 13.839 |
|  | B-F | 6.396 | 2.597 | 15.754 | 6.808 | 2.776 | 16.700 | 9.009 | 3.325 | 24.411 | 9.900 | 3.664 | 26.749 |
|  | C-F | 3.435 | 1.319 | 8.943 | 4.041 | 1.580 | 10.334 | 3.953 | 1.396 | 11.193 | 4.831 | 1.733 | 13.471 |
|  | D-F | 4.333 | 1.703 | 11.021 | 5.158 | 2.060 | 12.914 | 5.278 | 1.900 | 14.667 | 6.643 | 2.422 | 18.221 |
|  | E-F | 4.600 | 1.823 | 11.609 |  |  |  | 5.704 | 2.070 | 15.721 |  |  |  |
| Small airway restriction | A-F | 2.800 | 1.234 | 6.353 | 4.333 | 1.703 | 11.021 | 3.250 | 1.297 | 8.143 | 5.278 | 1.900 | 14.667 |
|  | B-F | 2.358 | 1.016 | 5.474 | 4.126 | 1.614 | 10.547 | 2.633 | 1.031 | 6.726 | 4.960 | 1.778 | 13.839 |
|  | C-F | 1.732 | 0.712 | 4.213 | 2.627 | 0.974 | 7.087 | 1.835 | 0.690 | 4.880 | 2.879 | 0.985 | 8.415 |
|  | D-F | 2.211 | 0.943 | 5.180 | 3.301 | 1.259 | 8.653 | 2.438 | 0.946 | 6.278 | 3.767 | 1.321 | 10.744 |
|  | E-F | 4.000 | 1.835 | 8.721 |  |  |  | 5.200 | 2.146 | 12.598 |  |  |  |
| Large airway obstruction | A-F | 4.126 | 1.614 | 10.547 | 3.782 | 1.605 | 8.916 | 4.960 | 1.778 | 13.839 | 4.621 | 1.781 | 11.991 |
|  | B-F | 2.888 | 1.083 | 7.706 | 2.407 | 0.965 | 6.002 | 3.215 | 1.110 | 9.313 | 2.650 | 0.973 | 7.218 |
|  | C-F | 2.223 | 0.802 | 6.159 | 1.515 | 0.561 | 4.095 | 2.379 | 0.794 | 7.126 | 1.568 | 0.536 | 4.588 |
|  | D-F | 2.476 | 0.907 | 6.759 | 2.407 | 0.965 | 6.002 | 2.689 | 0.909 | 7.954 | 2.650 | 0.973 | 7.218 |
|  | E-F | 1.600 | 0.542 | 4.720 |  |  |  | 1.653 | 0.521 | 5.244 |  |  |  |

There are no risks associated with lung function impairment among the children of Athi River schools in that season since RR and OR are less than 1 (CL 95%) and there is overlapping of null cases where RR and OR =1
